# Timely Diagnosis of Acyclovir-Induced Neurotoxicity During Acute Care Surgery Resuscitation: A Rare Complication of Common Herpes Zoster Treatment

**DOI:** 10.7759/cureus.92365

**Published:** 2025-09-15

**Authors:** Satoshi Ueno, Tsuyoshi Suzuki, Saki Takeda, Shigeru Maruhashi, Ken Iseki

**Affiliations:** 1 Department of Emergency and Critical Care Medicine, School of Medicine, Fukushima Medical University, Fukushima, JPN; 2 Department of Hepatobiliary Pancreatic and Transplant Surgery, School of Medicine, Fukushima Medical University, Fukushima, JPN; 3 Department of Forensic Medicine, School of Medicine, Fukushima Medical University, Fukushima, JPN

**Keywords:** acute care surgery, acyclovir, acyclovir-induced neurotoxicity, herpes zoster, toxicology, valacyclovir

## Abstract

Acyclovir-induced neurotoxicity is a serious adverse effect of acyclovir, which is widely utilized to treat herpes zoster, and presents with diverse neuropsychiatric symptoms. However, cases where psychiatric manifestations have resulted in self-inflicted abdominal injuries are extremely rare. Moreover, obtaining detailed medical and medication histories during trauma resuscitation is often difficult.

A 58-year-old man with a history of diabetes mellitus and chronic kidney disease on maintenance dialysis was found at home in a stuporous state after stabbing his abdomen and was transported in hemorrhagic shock. Emergency laparotomy showed transverse colonic and mesenteric injuries, which were treated with hemostasis and repair. During trauma resuscitation, it was discovered that the patient had ingested three days’ worth of valacyclovir, prescribed two days earlier for herpes zoster, inappropriately over a short period. The patient’s acute psychiatric manifestations were attributed to acyclovir-induced neurotoxicity in the absence of a psychiatric history. Hemodiafiltration was performed because the postoperative disturbance of consciousness persisted, and this resulted in rapid improvement. The patient was extubated on postoperative day 2, the neuropsychiatric symptoms resolved, and the patient was discharged on day 10. At admission, the whole blood acyclovir concentration was 6.45 μg/mL, and the concentration of its metabolite, 9-carboxymethoxymethylguanine, was 10.5 μg/mL, both of which were substantially elevated.

This case report underscores a rare presentation of Acyclovir-induced neurotoxicity in which neuropsychiatric symptoms led to self-inflicted abdominal injury and hemorrhagic shock. Even in the acute setting of trauma resuscitation, careful history-taking, including medication use, can be crucial for timely diagnosis and effective treatment. Clinicians should remain vigilant of acyclovir despite it being commonly prescribed for herpes zoster because of its potential to cause severe psychiatric manifestations, and sufficient patient education is essential to prevent its misuse.

## Introduction

Acyclovir (ACV)-induced neurotoxicity is a rare but serious adverse effect of ACV and valacyclovir (VACV), which is widely utilized to treat herpes zoster [1]. ACV and its metabolite 9-carboxymethoxymethylguanine (CMMG) are excreted renally. Thus, elevated concentrations and an increased neurotoxicity risk are noted, especially in elderly patients and those with renal impairment [2]. The clinical manifestations are diverse, including disturbances of consciousness, confusion, hallucinations, and abnormal behavior; however, they are nonspecific [1,3]. Additionally, viral encephalitis must be considered as a differential diagnosis because of its overlapping features [4]. Therefore, careful medical history-taking and evaluation of medication use are necessary for the diagnosis of ACV-induced neurotoxicity.

Although numerous ACV-induced neurotoxicity cases have been reported [1,4-7], neuropsychiatric symptoms resulting in self-inflicted injuries are extremely rare. Herein, we encountered a case where confusion due to ACV-induced neurotoxicity led to an abdominal stab wound and hemorrhagic shock. Despite the complexity of trauma resuscitation, ACV-induced neurotoxicity was identified as the underlying cause of the patient’s psychiatric manifestations. Blood purification therapy was promptly initiated, which resulted in a favorable outcome. This case highlights the criticality of obtaining a thorough medical history during acute trauma care and underscores the fact that even commonly prescribed medications for common diseases can lead to severe and life-threatening complications.

This case report contains anonymized personal information and did not require approval from an institutional ethics committee. Personal information was anonymized, and the patient and their family provided written informed consent for publication. This article was previously presented as a meeting abstract at the 37th Eastern Japan Regional Meeting of the Japanese Society of Clinical Toxicology on February 3, 2024.

## Case presentation

The patient was a 58-year-old man with a history of diabetes mellitus, hypertension, and chronic renal failure who had been on maintenance hemodialysis. The patient had no history of psychiatric illness. Two days before admission, the patient underwent routine hemodialysis in the morning and was prescribed VACV 500 mg once daily for three days for herpes zoster later that afternoon. The night before admission, the patient complained of malaise. In the early morning of the day of admission, the patient stabbed their abdomen in the kitchen at home and was found in an agitated and confused state by the family, who called for emergency services.

**Figure 1:**
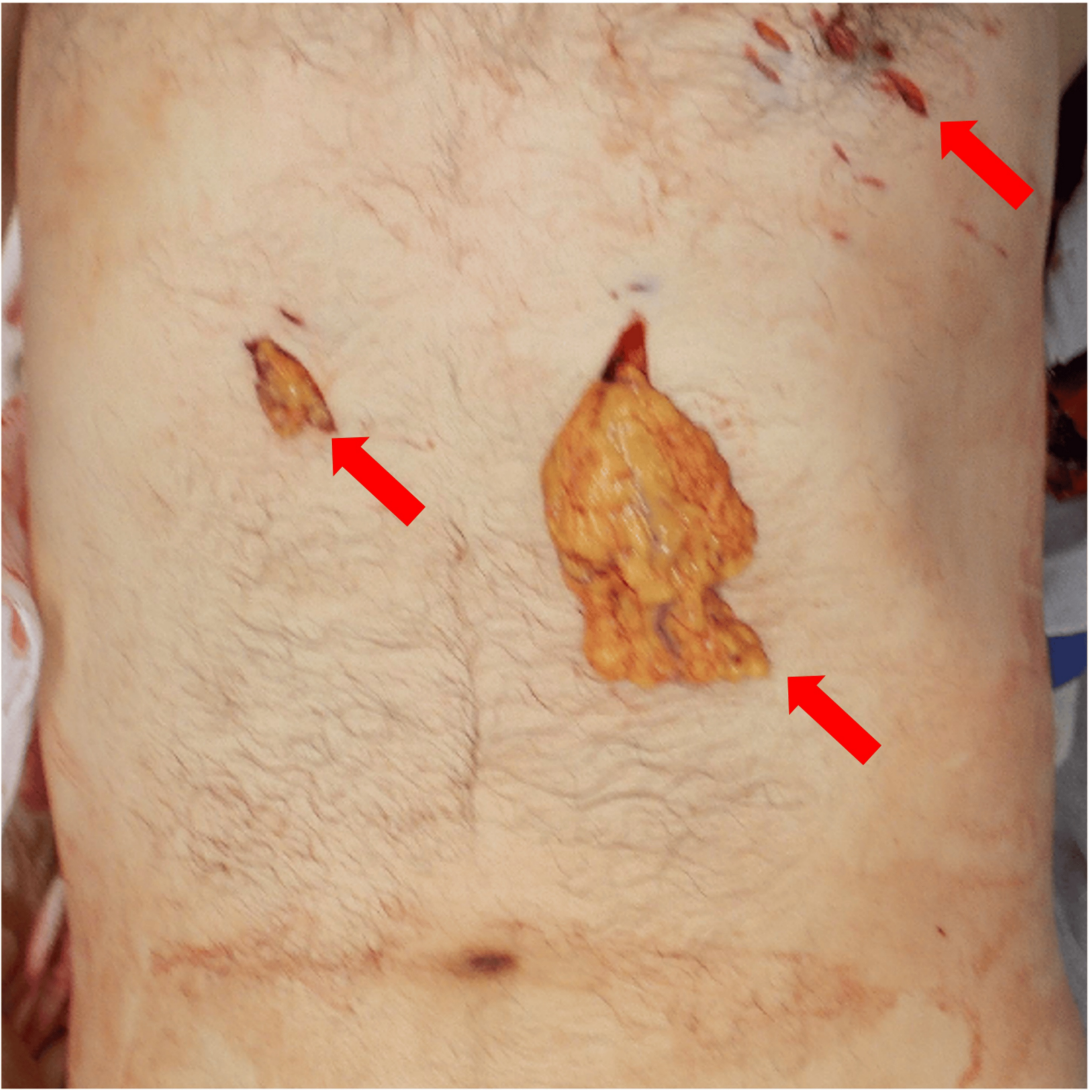
Clinical appearance on admission. Stab wounds were noted on the anterior chest and upper abdomen (indicated using arrows). Omental protrusion was noted in two abdominal wounds in the upper abdomen.

**Figure 2:**
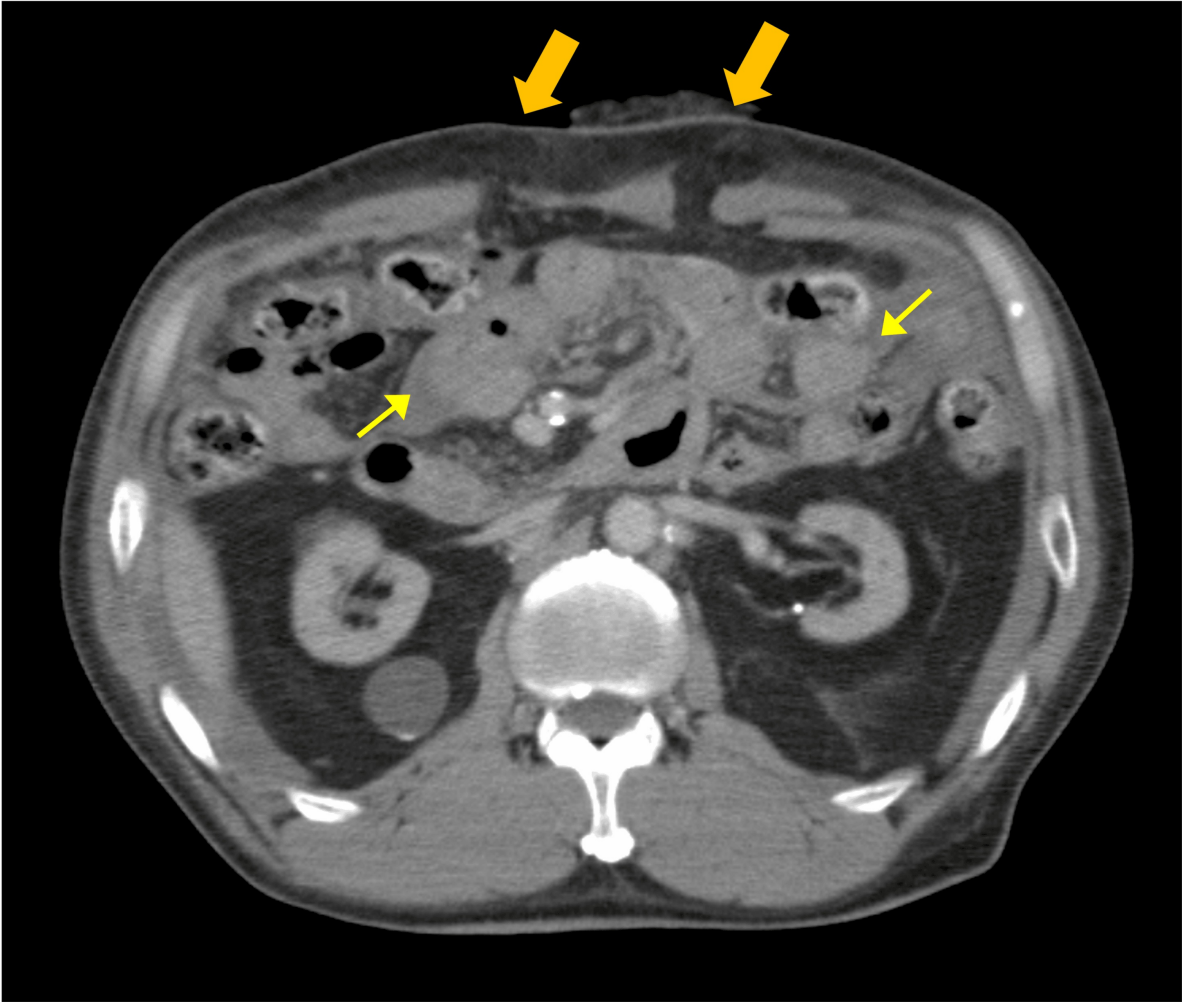
CT findings on admission. The CT showed a rectus abdominis laceration corresponding to the stab wound. Additionally, ascites was noted near the transverse colon, suggestive of bowel injury.

Upon contact with paramedics, the patient was agitated, with a blood pressure of 78/50 mmHg and a heart rate of 116 beats/minute, and was considered to be in hemorrhagic shock owing to an abdominal stab wound. On arrival at our hospital, the patient was pale, with a Glasgow Coma Scale score of E3V4M6. The patient remained agitated and had a blood pressure of 88/45 mmHg, heart rate of 90 beats/minute, and respiratory rate of 30 breaths/minute. Protrusion of the omentum through the abdominal wound was noted (Figure 1). Computed tomography showed ascites around the transverse colon, suggestive of bowel injury (Figure 2). Laboratory findings indicated severe anemia (hemoglobin: 8.0 g/dL; reference range, 11.6-14.8 g/dL) and lactic acidosis (lactate: 12.0 mmol/L; reference range, 0.4-2.0 mmol/L), consistent with hemorrhagic shock. Emergency laparotomy showed injuries in the transverse colon and mesentery, for which hemostasis and repair were performed. The operation lasted for 2 hours and 13 minutes, with an estimated blood loss of 2,530 mL.

**Figure 3:**
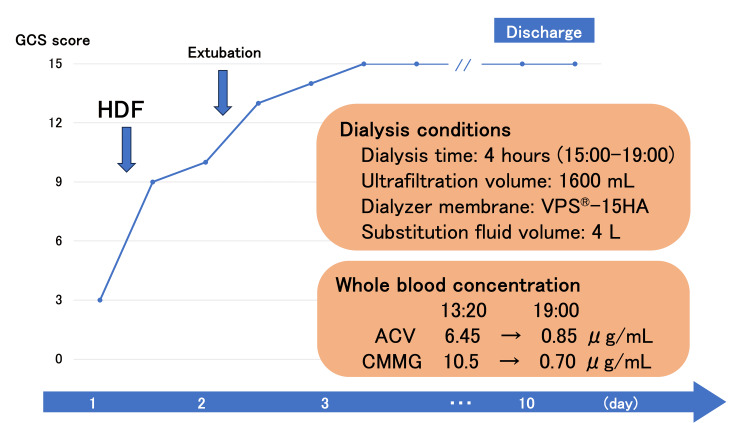
Clinical course of consciousness following admission and dialysis conditions. Hemodiafiltration was conducted immediately following surgery, leading to improved consciousness. Subsequent measurements showed reduced whole blood acyclovir and 9-carboxymethoxymethylguanine concentrations following dialysis. The dialyzer membrane used was VPS^®️^-15HA, a vitamin E-bonded polysulfone high-flux membrane. Image credit: All authors. GCS, Glasgow Coma Scale; HDF, hemodiafiltration; ACV, acyclovir; CMMG, 9-carboxymethoxymethylguanine

After admission to the intensive care unit, the patient was hemodynamically stable; however, an unexplained disturbance of consciousness persisted beyond what could be attributed to hemorrhagic shock alone. While the trauma treatment was ongoing, a detailed history was obtained from the patient’s family, which revealed that although three days of VACV had been prescribed, the patient had mistakenly taken all three prescribed doses within approximately 24 hours (one tablet after returning home, one before dinner, and another the following day after lunch). Based on this history, the psychiatric and consciousness disturbances were attributed to ACV-induced neurotoxicity. Thus, on the day of surgery, the patient underwent four hours of hemodiafiltration (HDF) for neurological symptom improvement. After HDF, the patient’s consciousness rapidly improved. Later analyses revealed that ACV and its metabolite, CMMG, markedly decreased following HDF on day 1: ACV concentrations 6.45 → 0.85 μg/mL and CMMG concentrations 10.5 → 0.70 μg/mL (pre-HDF at 13:20 vs. post-HDF at 19:00). Subsequently, the patient was extubated on postoperative day 2, transferred out of the ICU on day 3, and discharged on day 10. No recurrence of the neuropsychiatric symptoms was noted following discharge (Figure 3).

## Discussion

This case represents an extremely rare instance in which a patient without a psychiatric history developed confusion owing to ACV-induced neurotoxicity, leading to self-inflicted abdominal stab wounds and subsequent hemorrhagic shock. To the best of our knowledge, very few reports exist on the psychiatric manifestations of ACV-induced neurotoxicity that result in self-harm or severe trauma. In this case, detailed medical history enabled the early diagnosis of ACV-induced neurotoxicity, despite the challenges of trauma resuscitation. Although the patient underwent emergency surgery, prompt initiation of blood purification therapy resulted in rapid improvement in their neuropsychiatric symptoms. This outcome highlights the criticality of multidisciplinary teamwork, in which acute care surgeons and the emergency medical team collaborate to obtain a thorough history in parallel with trauma management.

Herpes zoster is a common disease in the elderly, with an estimated lifetime risk of one in three individuals by the age of 80 in Japan [8]. VACV and ACV are widely utilized for their treatment; however, several reports of ACV-induced neurotoxicity have been described, especially among elderly patients and those with renal impairment, warranting caution [1,4,5]. VACV is a prodrug of ACV with favorable gastrointestinal absorption and a longer half-life, which can lead to higher serum concentrations [9,10]. Although VACV offers the advantages of decreased dosing frequency and greater convenience than ACV, errors in dosage or administration may result in severe adverse effects [11]. Particularly in patients with renal failure, ACV concentrations are more likely to increase, necessitating dose adjustment and heightened vigilance for the development of ACV-induced neurotoxicity [5]. However, in recent years, with the aging of the population, cases of ACV-induced neurotoxicity have also been reported in patients without pre-existing renal failure [6]. This is attributed not only to the propensity of elderly individuals to develop renal impairment due to factors such as dehydration, but also to the fact that ACV itself can induce renal dysfunction [7]. Therefore, caution regarding ACV-induced neurotoxicity is required not only in patients with renal failure but also in elderly patients who may be predisposed to renal impairment. Furthermore, in elderly patients such as the present case, inadvertent short-term overuse can occur, emphasizing the necessity of thorough patient education at the time of prescription.

In the present case, a detailed history obtained in parallel with surgical management demonstrated clear evidence of inappropriate ACV use, which enabled the diagnosis of ACV-induced neurotoxicity and the timely initiation of blood purification therapy. The clinical manifestations of ACV-induced neurotoxicity, such as confusion, disturbance of consciousness, and hallucinations, are diverse and nonspecific. If the condition is not recognized, it may be overlooked and treatment delayed [3]. This risk is especially pronounced in the setting of acute trauma care, where thorough history-taking is often deprioritized, underscoring its importance even under such circumstances. ACV-induced neurotoxicity may occur at ACV concentrations >2 to 4 μg/mL and CMMG concentrations >6 to 10 μg/mL [12,13]. In our patient, the pre-dialysis ACV concentrations were 6.45 μg/mL, and the CMMG concentrations were likewise elevated at 10.5 μg/mL, consistent with those of previous reports. These findings supported the diagnosis; however, ACV concentrations could not be measured in real time in routine clinical practice. Moreover, in patients with impaired consciousness after herpes zoster, viral encephalitis must be considered in the differential diagnosis, as the clinical presentations are often similar. In viral encephalitis, fever, headache, and abnormal cerebrospinal fluid findings are typically observed; however, as in the present case, when impaired consciousness is present in combination with trauma-related inflammation, differentiation becomes challenging. MRI may also fail to show characteristic findings, and in patients exhibiting neuropsychiatric symptoms, prompt imaging can be difficult to perform [6]. Therefore, careful history-taking along with clinical assessment remains indispensable for establishing a diagnosis.

ACV-induced neurotoxicity treatment involves immediate discontinuation of the drug and initiation of blood purification therapy. Blood purification should be considered in patients presenting with severe neuropsychiatric symptoms, as rapid improvement can be expected [10,13]. This is because ACV has a low protein-binding rate and a small volume of distribution [9], allowing it to be efficiently removed by dialysis, with approximately 60% eliminated during a six-hour session [14]. In the present case, confusion persisted, although the patient’s vital signs stabilized after abdominal surgery, and hemodiafiltration was initiated. After a four-hour session, the ACV concentration reduced from 6.45 to 0.85 μg/mL, accompanied by substantial improvement in consciousness. The resolution of neuropsychiatric symptoms that could not be explained by trauma alone facilitated early extubation and contributed to a favorable postoperative course. Although some hesitation existed regarding the initiation of blood purification therapy immediately following emergency surgery, it proved to be useful as a treatment and diagnostically, in accordance with previous reports, by helping to distinguish ACV-induced neurotoxicity from viral encephalitis, which is often challenging to differentiate in clinical practice [4,6]. Thus, ACV-induced neurotoxicity should always be considered in patients with severe neuropsychiatric symptoms, and blood purification therapy should be actively pursued.

## Conclusions

Here, we report a case of ACV-induced neurotoxicity presenting with neuropsychiatric symptoms that led to abdominal self-injury. This case suggests that obtaining a detailed medical history, including medication use, even during trauma resuscitation, is crucial for timely diagnosis and appropriate therapeutic intervention. Additionally, although performed postoperatively, blood purification therapy proved effective for improving neuropsychiatric symptoms and distinguishing ACV-induced neurotoxicity from viral encephalitis. Although ACV and VACV are commonly prescribed for herpes zoster, clinicians should remain vigilant because of their potential to cause severe neuropsychiatric adverse effects, particularly in elderly patients with concomitant renal impairment, in whom elevated ACV serum concentrations increase the risk of developing ACV-induced neurotoxicity. Furthermore, sufficient patient education is necessary to prevent its misuse.
